# Antibody and cytokine response to *Cystoisospora suis* infections in immune-competent young pigs

**DOI:** 10.1186/s13071-018-2974-6

**Published:** 2018-07-04

**Authors:** Barbara Freudenschuss, Bärbel Ruttkowski, Aruna Shrestha, Ahmed Abd-Elfattah, Marc Pagès, Andrea Ladinig, Anja Joachim

**Affiliations:** 1 Institute of Parasitology, Department of Pathobiology, University of Veterinary Medicine Vienna, Veterinaerplatz 1, 1210 Vienna, Austria; 2 HIPRA, Amer, Spain; 3 University Clinic for Swine, Department for Farm Animals and Veterinary Public Health, University of Veterinary Medicine Vienna, Veterinaerplatz 1, 1210 Vienna, Austria

**Keywords:** Coccidiosis, Pig, Immunity, Antibody, Cytokine, Immunization, Stimulation, T helper 2

## Abstract

**Background:**

To date, investigations on the immune response to *Cystoisospora suis* infections focused on suckling piglets, the age group clinically most affected. Actively immunizing piglets is unfeasible due to their immature immune system and the typically early infection in the first days after birth. Therefore, understanding and possibly enhancing the immune response of immune-competent animals is the prerequisite to develop a passive immunization strategy for piglets which currently rely on very limited treatment options.

**Methods:**

To investigate antibody and cytokine responses of immune-competent animals and the impact of the oral immunization protocol on their immune response, growers with unknown previous exposure to *C. suis* (10–11 weeks-old) were infected one or three times with different doses (600 and 6000 or 200 and 2000, respectively) of *C. suis* oocysts, and compared to uninfected controls. Oocyst excretion was evaluated, and blood and intestinal mucus antibody titers were determined by IFAT. Systemic production of Th1, Th2, inflammatory and regulatory cytokines was determined in different immune compartments at mRNA and (after stimulation with a recombinant merozoite-protein) at protein level by PCR and multiplex fluorescent immunoassay, respectively.

**Results:**

Infection generated significantly increased serum IgA and IgG levels against *C. suis* sporozoites and merozoites, irrespective of infection mode, with IgG against merozoites showing the strongest increase. No clinical signs and only occasional excretion were observed. The systemic cytokine response to *C. suis* was only weak. Nonetheless, in white blood cells, IL-4, IL-6 and IL-10 mRNA-levels significantly increased after infection, whereas IFN-ɣ, IL-2 and TGF-β expression tended to decrease. In mesenteric lymph nodes (MLN), IL-10 and TNF-α levels were elevated while splenic cytokine expression was unaltered upon infection. Stimulated MLN-derived lymphocytes from infected pigs produced slightly more IL-12 and less IFN-α than controls.

**Conclusions:**

An infection and a subsequent systemic immune response can be induced in immune-competent animals by all evaluated infection models and growers can be used as models to mimic sow immunizations. The immune response to *C. suis*, although mild and with considerable variation in cytokine expression, was characterized by a Th2-associated and regulatory cytokine profile and antibody production. However, none of the parameters clearly stood out as a potential marker associated with protection. Antibody titers were significantly positively related with oocyst excretion and might thus serve as correlates for parasite replication or severity of infection.

**Electronic supplementary material:**

The online version of this article (10.1186/s13071-018-2974-6) contains supplementary material, which is available to authorized users.

## Background

The coccidian species *Cystoisospora suis* (syn. *Isospora suis*) occurs worldwide at high prevalences, with neonates and suckling piglets being specifically susceptible to clinical disease [1–4]. Oocysts as resistant environmental stages, together with a high morbidity rate, make neonatal porcine cystoisosporosis one of the major causes of diarrhea in piglet production, resulting in impaired weight gain and subsequently reduced, uneven weaning weights [5, 6], and thus inflicting serious financial losses to the farming industry [7–9].

Toltrazuril is currently the only compound available to successfully contain cystoisosporosis [10–12]. However, toltrazuril resistance has recently been reported for the first time in a field isolate of *C. suis* [13], and the emergence of more widespread resistance as described in avian coccidia [14, 15] has to be expected. Moreover, the potential hazard of drug residues in animal products and environment is of growing concern to the public [16–18]. Canada, for example, rescinded the submission process of Baycox® in 2005 because carcinogenic properties of toltrazuril could not be ruled out [19], leaving farmers without satisfying alternatives. Consequently, the necessity of new strategies to combat this parasitic disease, possibly targeting the immune system, has dramatically increased.

The involvement of humoral or cellular immune components and their protective role in the case of *C. suis* are not yet fully understood. The cellular immune system is considered a key player in controlling infections with mammalian and avian coccidia [4, 20–24] and an involvement has also been shown in piglets infected with *C. suis* [25–27], although only limited data on cytokine responses of the involved cell populations are available. On the other hand, humoral immune activity has been demonstrated in *C. suis* [28, 29] and other coccidia [30–33] but its contribution to the development of protective immunity remains to be resolved [4, 9, 20, 22, 23, 30, 33].

Until now, investigations on the immune response to *C. suis* focused primarily on suckling piglets as the most affected age group. Yet the immune mechanisms of older, immune-competent pigs are of interest, particularly with regard to the development of a passive immunization strategy for piglets. Immunizing neonates with a live vaccine similar to poultry [34, 35] is not possible as their immature immune system [36–38] and interfering maternal immunity would not allow for an adequate immune response at this early stage of life [39, 40] when the infection is most relevant [1, 7]. To date, only few data are available on the immune response of immunologically mature animals. Worliczek et al. [41] demonstrated an antigen-specific IFN-ɣ response of splenocytes from weaners upon challenge, and Schwarz et al. [29] showed an increase of specific antibodies in blood serum and colostrum of pregnant sows after high-dosed infections, indicating that the infection of immune-competent animals can induce an immune response, despite the absence of oocyst shedding [29, 41]. Moreover, superinfection of pregnant sows was followed by a milder course of disease in their experimentally infected offspring. However, a sufficient protection of piglets against clinical cystoisosporosis was not achieved, and also the routine administration of oocysts to sows in such high doses is not practical.

This study aimed to gain a better understanding of the immune-competent host’s response to *C. suis* and we therefore investigated antibody- and cytokine-related aspects of the immune response of immunologically mature grower pigs with unknown previous contact to *C. suis*. We also evaluated whether intensity and character of their immune response depend on the protocol of oral antigen delivery and whether a potential immunization model for sows can be identified which could enhance their immune response and subsequently improve passive protection of piglets. Lastly, we sought to identify potential markers associated with immunization upon experimental infection.

## Methods

### Study animals

A total of 50 female crossbred growers from a conventional producer in Lower Austria with an unknown history of coccidiosis were used. Animals were 10–11 weeks-old at the beginning of the trial and clinically examined and weighed upon arrival. Their status of immunity against *C. suis* was unknown. Those animals that were going to be infected with *C. suis* oocysts were allocated to four different groups (Table 1) according to body weight ranking using a pre-set randomization scheme. Three separate infection trials with pigs from at least two litters per trial allocated to the respective infection groups were carried out to receive final group sizes of 10. Animals were kept on straw with daylight under conventional conditions at the facilities of the Institute of Parasitology, University of Veterinary Medicine Vienna, and arrived one week before the start of the trial for acclimatization. Uninfected control animals were sampled in an independent trial including pigs from two litters; they were housed in a biosafety unit of the Clinic for Swine, University of Veterinary Medicine Vienna, to prevent infections with coccidia, and arrived one day before the start of the trial. All animals had access to water *ad libitum* and were fed once daily with a commercial pig diet. The day of (first) infection was defined as study day (SD) 1.

**Table 1:** Groups and infection regimes for growers infected with oocysts of *C. suis*

Group	Infection dose	Days of infection	No. of animals
Single High (SH)	6000 oocysts *per os*	SD 1	10
Single Low (SL)	600 oocysts *per os*	SD 1	10
Trickle High (TH)	3 × 2000 oocysts *per os*	SD 1, SD 8, SD 15	10
Trickle Low (TL)	3 × 200 oocysts *per os*	SD 1, SD 8, SD 15	10
Control (C)	1 ml tap water *per os*	SD 1	10

*Abbreviation*: *SD* study day

### Parasite material and experimental infection

Oocysts of *C. suis*, strain Wien-I [10], obtained during previous experimental infections of piglets, were isolated from feces, brought to sporulation and stored as described [28]. Oocysts were washed with tap water prior to infection. Animals were infected with different oral doses of sporulated oocysts at different frequencies (infected groups) or sham-treated with tap water (Control) on SD 1 (see Table 1 for details).

### Sample collection

Individual fecal samples were taken on the day of arrival, on SD 1 and daily from SD 6–29 and examined for the presence of *C. suis* oocysts. Serum and whole blood samples were collected from the jugular vein on SD 1, 8, 15 and 22, and from the heart on SD 29 (after anesthesia) using a needle (18 G) and syringes (Primavette® V 10 ml, KABE Labortechnik GmbH, Nümbrecht, Germany). On SD 29 animals were sacrificed by exsanguination following deep general anesthesia [10 mg/kg body weight (BW) of ketamine and 1.2 mg/kg BW of azaperone]. Spleen and small intestines including mesenteric lymph nodes (MLN) were isolated immediately post mortem and kept in cold, sterile PBS (Gibco™, Thermo Fisher Scientific, Waltham, MA, USA) until processing. Hematology and white blood cell counts (Additional file 1: Table S1) were performed on SD 1 and 29 by the Central Laboratory, Department of Pathobiology, University of Veterinary Medicine Vienna.

### Sample processing

#### Blood

Serum was obtained by centrifugation of coagulated blood samples (10 min at 1500× *g*) and stored at -20 °C until further use. White blood cells were purified from 4 ml of heparinized blood within two hours of sampling by erythrolysis using Buffer EL (Qiagen, Hilden, Germany), following the manufacturer’s instructions. Homogenized cell lysates were either stored at -80 °C until further processed or directly subjected to total RNA extraction.

#### MLN and spleen

MLN were isolated from the small intestinal area and separated from tissue; spleen was minced into small pieces and capsular tissue was removed. Lymphocytes were isolated from MLN under sterile conditions as described previously [26] and cells were suspended in RPMI 1640 (Gibco™, Thermo Fisher Scientific) supplemented with 40% FCS and 10% dimethyl sulfoxide and stored in liquid nitrogen. Additionally, MLN and spleen samples were snap frozen in liquid nitrogen and stored at -80 °C for later extraction of nucleic acids.

#### Intestines

Small intestines were arranged in a meandering pattern; the part defined as the mid-section of the jejunum and considered most suitable for further studies [42] was sampled. Ten centimeter long segments were sampled for the retrieval of mucus (*n* = 3 pieces) according to Schwarz et al. [28]. Supernatants containing mucus antibodies were stored at -20 °C until serology was performed. One centimeter long segments were sampled for preservation in 10% buffered formalin and colorless Neg-50™ Frozen Section Medium (Richard-Allan Scientific, Kalamazoo, MI, USA), respectively. The latter were immediately snap frozen in liquid nitrogen and stored at -80 °C. Transverse sections of formalin-fixed tissues were embedded in paraffin, cut and stained with hematoxylin and eosin (H&E) according to standard procedures.

### Serology

Titers of immunoglobulin (Ig) G and IgA against *C. suis* sporozoites and merozoites as antigens were measured in blood serum and intestinal mucus samples using an immunofluorescence antibody test (IFAT). Sporozoites were derived by excystation of oocysts as described by Worliczek et al. [43] with minor changes. Briefly, purified oocysts were vortexed with 200 μl sodium hypochlorite (12%) and subsequently incubated at 4 °C for 15 min. After being washed with PBS the resuspended pellet was vortexed with Precellys® glass beads (peqlab, VWR International GmbH, Erlangen, Germany) 3 times for 20 s. Parasites were transferred to a 15 ml tube, incubated in 2 ml of a taurocholate/trypsin solution (Sigma-Aldrich, Vienna, Austria; 7.5 and 2.5 g/l PBS, respectively) for 60 min (37 °C, 5% CO_2_) and subsequently washed with PBS*.* Merozoites were grown in intestinal porcine epithelial cells (IPEC-J2) [43] and harvested by collecting cell culture supernatants 6–10 days after infection of IPEC-J2 with sporozoites. 10-dot slides (Medco Diagnostika GmbH, Hengersberg, Germany) were coated with either of the parasite stages diluted in PBS. Slides were dried overnight at 40 °C, fixed in -20 °C cold acetone for 10 min and stored at -80 °C for later use. IFAT was performed according to Schwarz et al. [28]. In brief, slides were incubated with serum or intestinal mucus samples in serial dilutions starting at 1:20, washed and subsequently incubated with fluorescein-labeled antibodies [goat anti-swine IgG (Kirkegaard & Perry Laboratories, Gaithersburg, MD, USA) diluted 1:150 in PBS or goat anti-swine IgA (Bethyl Laboratories, Montgomery, TX, USA) diluted 1:1000 in PBS]. Slides were washed, covered with PBS/glycerin (1:10) and a coverslip, and titers were assessed under fluorescence. Positive and negative controls were included in each IFAT. Titers were converted to numerics for statistical calculations, starting with 1 representing a titer of 1:20.

### Quantification of cytokine mRNA expression in white blood cells and lymphatic tissues

Relative quantitative real-time PCR (qPCR) was performed to quantify cytokine transcription in white blood cells, MLN and spleen. For this, total RNA was isolated from homogenized leukocyte lysates (see above) and MLN and spleen tissue using a QIAamp® RNA Blood Mini Kit (Qiagen) including the optional on-column DNase digestion (Qiagen) according to the manufacturer’s instructions. The amount and integrity of extracted total RNA were evaluated using a NanoDrop® 2000 (Thermo Fischer Scientific) and agarose gel electrophoresis, respectively. For cDNA synthesis 1 μg of total RNA was reversely transcribed using an iScript™ cDNA Synthesis Kit (Bio-Rad Laboratories, Hercules, CA, USA). In four cases, however, less RNA (200 ng) had to be used due to low yields. qPCR was carried out on a Mx3000P thermal cycler (Agilent Technologies, Santa Clara, CA, USA) using a reaction mix with a total volume of 10 μl, containing the following components: 5 μl SsoAdvanced™ Universal SYBR® Green Supermix (Bio-Rad Laboratories), forward and reverse primers (see Table 2 for details on primers and targets) and 0.75 μl of 1:5 diluted cDNA. Thermal cycles composed of an initial 3 min denaturation phase at 95 °C followed by 40 cycles of 95 °C for 15 s (IL-12: 94 °C for 20 s), annealing at primer-specific temperatures (Table 2) for 30 s, and extension at 72 °C for 30 s (IL-12: 20 s), and a melting curve analysis afterwards. Samples were amplified in duplicates. Non-template controls were included in every run, as well as interplate calibrators (IPC) to normalize C_q_ values measured on different plates and thus correct for inter-run-variation. Efficiency of primers was determined by five-fold dilution series of a cDNA pool; agarose gel electrophoresis and melting curve characteristics were used to evaluate their specificity. Relative target gene expression was calculated applying the software GenEx 6 (MultiD, Goteborg, Sweden), including correction of qPCR data for primer efficiency, interplate calibration and normalization to the expression of reference genes OAZ1 and RPL4 (Table 2). Data were converted to a log_2_ scale for statistical analyses. For relative quantification, samples were expressed relative to a calibrator, either their corresponding samples from SD 1 (to analyze group differences) or the sample with the lowest expression level (to analyze changes over time), which were scaled to 0.

**Table 2:** Targets and details of primers used for qPCR

Gene	Accession number	Primer sequence 5′-3′	Product size (bp)	Annealing temp. (°C)	Primer conc. (nM)	Primer efficiency (%)	Source
RPL4^a^	XM_005659862.1	F: ATGCAAAGACAATGCGCCGA	127	60	400	101.7	This study
		R: ACGGGCTTCTTGTCTGGAAC					
OAZ1^a^	NM_001122994.1	F: CAATAGCTGCCTCTACATCGA	134	60	400	100.2	[27]
		R: GGTTCTTGTGGAAGCAAATGAAG					
IFN-ɣ	NM_213948	F: GCTCTGGGAAACTGAATGAC	167	60	300	103.9	[75]
		R: TCTCTGGCCTTGGAACATAG					
IL-12p35	NM_213993.1	F: CATGTGTCCGCTGCGCAACCT	98	69	250	99.5	[53]
		R: GCTGTGGTTGCAGGGAGGCTC					
IL-2	NM_213861	F: GCCATTGCTGCTGGATTTAC	159	62	200	105.0	[75]
		R: CCCTCCAGAGCTTTGAGTTC					
TNF-α	NM_214022	F: ACTGCACTTCGAGGTTATCGG	118	60	300	98.9	[76]
		R: GGCGACGGGCTTATCTGA					
IL-1β	NM_001005149	F: AGAAGAGCCCATCGTCCTTG	139	60	300	99.5	[75]
		R: GAGAGCCTTCAGCTCATGTG					
IL-27	EST BP439244	F: GCCCGCCACTTTGCTGAATC	152	60	300	94.7	[75]
		R: GGGCGAAGTGTCATGGAGAG					
IL-6	NM_214399	F: ATCAGGAGACCTGCTTGATG	177	60	300	101.7	[75]
		R: TGGTGGCTTTGTCTGGATTC					
IL-4	NM_214123.1	F: CAACCCTGGTCTGCTTACTG	173	60	300	91.9	[77]
		R: CTTCTCCGTCGTGTTCTCTG					
IL-10	NM_214041	F: CCGAAGGCAGAGAGTGATGGG	111	60	300	103.9	[78]
		R: ACAGGGCAGAAATTGATGACAGC					
TGF-β	NM_214015	F: GAAGCGCATCGAGGCCATTC	162	60	300	104.9	[75]
		R: GGCTCCGGTTCGACACTTTC					

^a^Reference genes for normalization

*Abbreviations*: *F* forward, *R* reverse, *RPL4* ribosomal protein L4, *OAZ* ornithine decarboxylase antizyme, *IFN* interferon, *IL* interleukin, *TGF* transforming growth factor

### In vitro stimulation of lymphocytes

Cells isolated from MLN were thawed and stimulated in duplicates with a recombinant merozoite protein (rCSUI_005805) recently characterized by Shrestha et al. [44] at a final concentration of 10 μg/ml. Cells were incubated in 48-well tissue culture plates at a density of 1 × 10^6^/well in a volume of 250 μl culture medium (RPMI 1640 with 10% FCS, 2 mM L-glutamine, 100/ml penicillin and 0.1 mg/ml streptomycin; Gibco™, Thermo Fisher Scientific) for 30 h at 37 °C with 5% CO_2_. In parallel, lymphocytes stimulated with Concanavalin A (5 μg/ml; Sigma-Aldrich) or incubated in culture medium only served as positive and negative controls, respectively. Supernatants were analyzed for various cytokines and chemokines (IL-1β, IFN-α, IFN-ɣ, IL-10, IL-12p40, IL-4, IL-8, CCL-2) by multiplex fluorescent microsphere immunoassay (FMIA) as described elsewhere [45].

### Quantification of parasite DNA in tissue

To study the intestinal and extraintestinal presence of stages of *C. suis*, DNA was extracted from jejunum (embedded in Neg-50™ Frozen Section Medium) and from snap-frozen MLN and spleen samples using a peqGOLD Tissue DNA Mini Kit (peqlab, VWR International GmbH, Erlangen, Germany), following the manufacturer’s instructions. Prior to DNA isolation, samples were slightly thawed on ice, several sections were excised at different locations, pooled, and the Neg-50™ Frozen Section Medium was removed from intestinal tissue by washing it three times in 1 ml of LiChrosolv® Water (Merck, Darmstadt, Germany). Extracted DNA was diluted 1:10 and qPCR was performed to quantify the *C. suis* genome by targeting the large subunit rRNA gene (GenBank: AF093428.1) as previously described by Shrestha et al. [44]. Samples were run in duplicates together with controls.

### Statistical analyses

Statistical calculations were performed with RStudio version 0.99.896 (RStudio Team, 2016). Group differences were analyzed by applying an ANOVA to data with normal distribution and variance homogeneity or, in the opposite case, either a Wilcoxon or (when comparing more than two samples) a Kruskal-Wallis rank sum test. In case of significant omnibus tests, *post-hoc* tests for multiple comparisons were performed (parametric Tukey or non-parametric Conover tests), and *P*-values were adjusted after Bonferroni. To analyze changes of values over time, parametric ANOVA or non-parametric Friedman rank sum tests, followed by multiple pairwise comparisons (t-tests and tests after Conover, respectively), were employed. Relationships between selected parameters were calculated by computing Spearman’s rank correlation coefficients. *P*-values ≤ 0.05 were considered significant.

## Results

### Clinical and fecal examination

Low levels of oocyst excretion occurred in every group infected with *C. suis* while uninfected control animals did not shed oocysts throughout the study. Highest numbers of shedders and excretion days were found in group SH where 70% of the pigs excreted oocysts on a total of 17 days (Table 3); however, statistics did not reveal significant differences in these parameters between infected groups. One animal (TH 5) excreted oocysts before infection and was thus treated with toltrazuril once (Baycox® 5% oral suspension, 20 mg/kg BW). It remained in the study but was excluded from analyses and comparisons involving the parameter “oocyst excretion”. None of the pigs developed diarrhoea during the study period. One animal of group SH developed a fever and general depression during the study and was treated with enrofloxacin, an antibiotic without anticoccidial effect, for three days.

**Table 3:** Results of coproscopical examinations on the day of (first) infection and from study day 5 to 29. × indicates the days of oocyst excretion. Only animals with at least one day of shedding and only study days with at least one excreting animal are listed

Animals	Study days																				
	1	8	9	10	11	12	13	14	15	16	17	18	19	20	21	22	23	24	25	27	29
SL 8														×							×
SL 10																			×		
SH 1											×	×									
SH 3													×	×	×						
SH 4															×	×					
SH 5								×													
SH 7						×					×					×					
SH 9				×					×												
SH 10												×		×				×	×		
TL 3								×				×	×	×			×				
TL 5														×							
TL 7				×	×		×	×	×		×										
TL 9																				×	
TH 5	×																				
TH 8			×													×					
TH 9										×	×										
TH 10		×	×																		

*Abbreviations*: *SL* Single Low, *SH* Single High, *TL* Trickle Low, *TH* Trickle High

### Hematology and white blood cell count

Most parameters were within normal limits except for a few mild deviations observed in all five groups. Statistical comparisons were only performed for parameters which deviated from their expected reference range at least once in at least one animal. Significant differences between infected and uninfected animals and over time in the respective groups were found for several hematological (hemoglobin, hematocrit) parameters and white blood cell populations (total leucocytes, lymphocytes, monocytes and segmented neutrophils/μl whole blood) (Additional file 1: Table S2).

### Histopathology of jejunum

H&E stained sections of the mid-jejunum were viewed for histopathological changes characteristic for *C. suis* infections [8, 46]. However, neither characteristic alterations nor differences between infected and control animals could be observed.

### *Cystoisospora suis* specific antibodies in blood serum and intestinal mucosa

**Fig. 1:**
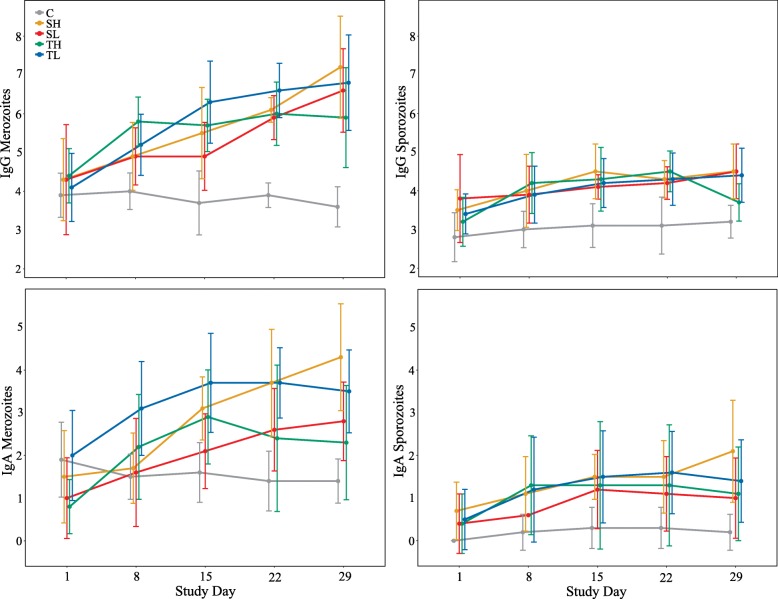
Development of mean blood serum antibody titers (IgG, IgA) against *C. suis* sporozoites and merozoites after infection. Titers were converted to numerics, starting with 1 representing a titer of 1:20, followed by 2 representing 1:40, and so on. Means and standard deviations are given for each group on each study day. Study day 1: day of (first) infection. *Abbreviations*: SL, Single Low; SH, Single High; TL, Trickle Low; TH, Trickle High; C, Control

*Cystoisospora suis*-specific IgG and IgA against both merozoites and sporozoites could be detected in all serum samples from SD 1 onwards, with significant differences between infected animals and sham-treated controls in all antibody classes against both antigens. IgG titers of groups SL, SH, TL and TH progressively increased after the (first) infection and remained at significantly elevated levels until the end of the study (SD 29) when compared to basal values prior to infection (Fig. 1 and Additional file 1: Table S3). IgG against merozoites reached significantly elevated values at 1 (groups TL and TH), 2 (SH) and 3 (SL) week(s) post (first) infection when compared to the non-infected group C and remained significantly higher until SD 29. Differences between infected groups were only observed on SD 8 and 15 with significantly higher titers in trickle than in single infected animals (see Fig. 1 and Additional file 1: Table S4). By SD 8, groups SL, SH and TH exhibited significantly higher titers of IgG against sporozoites than group C while for TL significantly increased levels were first recorded on SD 15. Except for TH, titers remained significantly higher than those of group C until the end of the study (Additional file 1: Table S4 and Fig. 1). Levels of IgA were relatively low compared to those of IgG, nevertheless titers against merozoites significantly increased over time in all infected groups, whereas for IgA against sporozoites a statistically confirmed increase over time was only seen for groups SL and SH (Fig. 1 and Additional file 1: Table S3). When compared to group C, significantly increased levels of IgA against merozoites were first observed in group TL from SD 8 onwards while SH and SL reached significantly elevated levels by SD 15 and 29, respectively. Titers of TH were higher than those of C, but a statistical difference was only seen on SD 15. Significant differences between infected groups were found for SL and TL on SD 8 and 15 and for SH and SL and SH and TH on SD 29 (Additional file 1: Table S4 and Fig. 1). IgA titers against sporozoites were higher in infected animals (Fig. 1) but significant differences to uninfected controls were only seen for SH and TL on SD 15 and 22 and for SH on SD 29 (Additional file 1: Table S4).

No significant differences were detected between groups SL, SH, TL and TH with regard to titer increase over time (from SD 1 to 22 or 29, respectively) in any of the evaluated antibody classes (data not shown).

In jejunal mucus, only IgG titers against merozoites were present but only in low amounts, with significantly higher levels in infected animals (*W* = 330.5, *P* = 0.001), specifically in groups SH and TH (*χ*^2^ = 11.261, *df* = 4, *P* = 0.029 and 0.044, respectively), when compared to uninfected ones.

The analysis of relationships between antibody levels and oocyst excretion revealed significant positive correlations between the number of days with excretion and the increase of serum IgG and IgA against both *C. suis* stages in the course of the study (Table 4). Positive correlations with the number of excretion days were also detected for serum titers of single study days: for IgG against merozoites on SD 15, for IgA against merozoites on SD 15, 22 and 29 and for both IgG and IgA against sporozoites on SD 29 (Table 4). Regarding IgG titers against merozoites in intestinal mucus, a similar trend could be observed but without statistical significance (*ρ* = 0.152, *P* = 0.292).

**Table 4:** Relationships between serum antibody titers and the number of days with oocyst excretion. Relationships are given as Spearman’s rank correlation coefficient *ρ* (in parentheses: *P*-values). Excretion days were correlated with IFAT results of all study days and with the titer development over time (study days 1 to 22 and 29, respectively). Significant correlations (*P* ≤ 0.05) are indicated in bold

Study day	Merozoites		Sporozoites	
	IgG	IgA	IgG	IgA
1	-0.176 (0.221)	-0.221 (0.123)	-0.009 (0.952)	0.121 (0.404)
8	0.108 (0.454)	-0.054 (0.711)	0.039 (0.787)	0.128 (0.375)
15	0.385 (**0.006**)	0.326 (**0.021**)	0.256 (0.072)	0.182 (0.206)
22	0.241 (0.091)	0.306 (**0.031**)	0.261 (0.068)	0.266 (0.062)
29	0.167 (0.247)	0.315 (**0.026**)	0.333 (**0.018**)	0.353 (**0.012**)
1–22	0.405 (**0.004**)	0.416 (**0.003**)	0.215 (0.138)	0.253 (0.079)
1–29	0.311 (**0.030**)	0.448 (**0.001**)	0.293 (**0.041**)	0.370 (**0.009**)

### Cytokine response to *C. suis* infections

To investigate a potential systemic involvement of the cellular immune system in *C. suis* infections, the mRNA expression of selected pro-inflammatory (IFN-ɣ, IL-12, IL-2, TNF-α, IL-6, IL-27, IL-1β) and regulatory (IL-4, IL-10, TGF-β, IL-27) cytokines was measured in cells from three immune compartments: white blood cells, lymphocytes from MLN and splenocytes. No data could be obtained from three blood samples (TL 7/SD 8, SH 5/SD 29 and C 10/SD 29) due to an insufficient amount of extracted RNA.

#### White blood cells

**Fig. 2:**
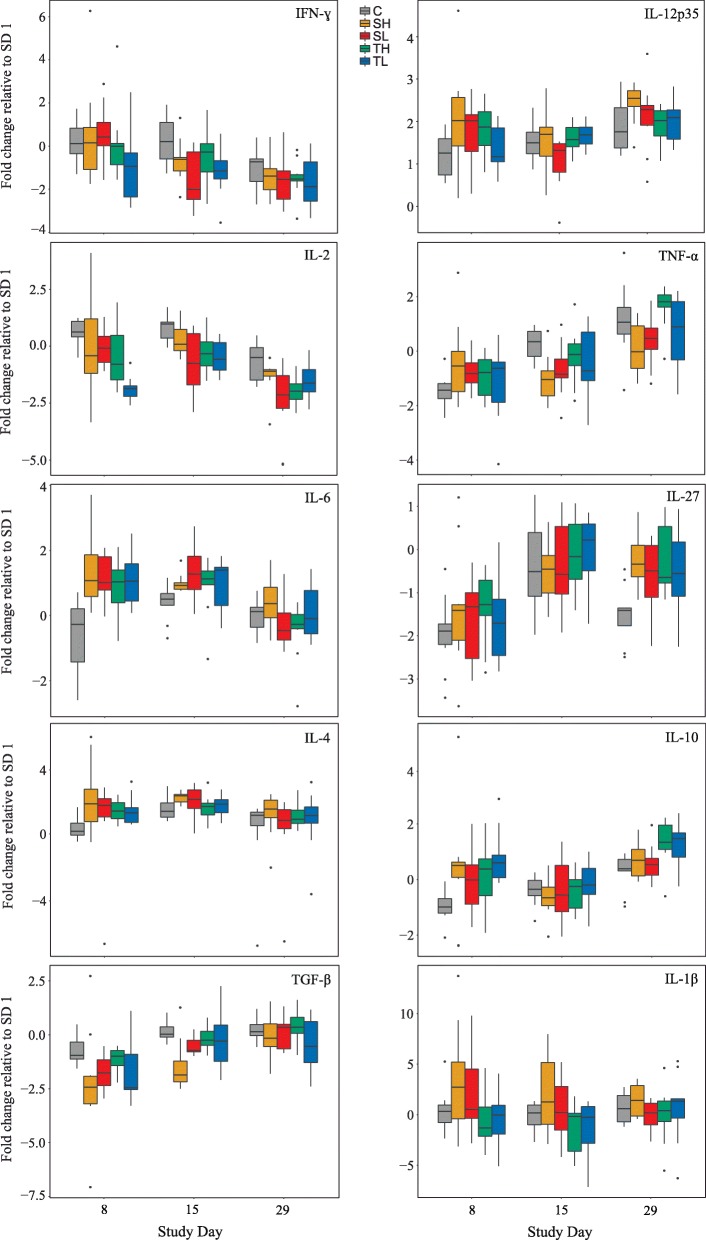
Cytokine mRNA expression of white blood cells after infection with *C. suis* and sham-treatment, respectively. Each sample from study day 8, 15 and 29 was normalized to its corresponding sample from study day 1 [i.e. before (first) infection]. Thus, fold expression values are given relative to values of this time point. Length of boxes indicates the interquartile range; the embedded line represents the median. Whiskers extend to the largest and smallest values still within 1.5 times the length of the interquartile range from the upper and lower quartiles, respectively. Data lying outside this range are plotted individually as dots. *Abbreviations*: SL, Single Low; SH, Single High; TL, Trickle Low; TH, Trickle High; C, Control

For most cytokines, expression patterns were similar between infected groups; however, relative mRNA quantities showed a high degree of variation between individuals, also within the same group. Expression of some cytokines also varied to a certain extent in uninfected animals between study days (Fig. 2). For details on the change of expression levels over time, see Additional file 1: Table S5. Levels of TNF-α, IL-10 and IL-2 differed between groups on SD 1 already, i.e. before infection (Additional file 1: Table S6), but these discrepancies are compensated for by the paired analysis (normalization to SD 1) applied here.

The infection with *C. suis* induced a significant decline of IFN-ɣ mRNA expression from SD 1 to 29 (Fig. 2). As a result, IFN-ɣ mRNA levels of infected animals were significantly lower on SD 15 compared to those of uninfected animals (*W* = 94, *P* = 0.009).

IL-12p35 levels significantly increased over time after infection, while they remained relatively constant in group C. Significantly more IL-12 mRNA was thus expressed in infected animals on SD 8 (*W* = 281, *P* = 0.032) (Fig. 2). Considering single- and trickle-dose infected animals separately revealed that groups which received a single infection contributed the most to this increase (*χ*^2^ = 32.886, *df* = 8, *P* = 0.047, compared to C).

Infected animals taken together expressed significantly less IL-2 mRNA on every study day (SD 8: *W* = 85, *P* = 0.005; SD 15: *W* = 60, *P* < 0.001; SD 29: *W* = 76, *P* = 0.007), with significantly lower levels in trickle infected animals on SD 8, 15 (*χ*^2^ = 65.63, *df* = 8, *P* < 0.001) and 29 (*χ*^2^ = 65.63, *df* = 8, *P* = 0.021) and in groups with single infections on SD 15 (*χ*^2^ = 65.63, *df* = 8, *P* = 0.009), when compared to the uninfected group (Fig. 2). Additionally, IL-2 was significantly less expressed in trickle than in single infected animals on SD 8 (*χ*^2^ = 65.63, *df* = 8, *P* = 0.014).

TNF-α mRNA expression displayed strong variations both in infected and uninfected animals (Fig. 2). Nevertheless, it slightly increased in trickle infected animals from SD 1 to 29, resulting in significantly elevated levels compared to single-dose infected groups at the end of the study (*χ*^2^ = 57.271, *df* =8, *P* = 0.018). On SD 8, TNF-α expression levels of infected animals were significantly elevated compared to controls (*W* = 285, *P* = 0.025) while they were significantly reduced on SD 15 (*W* = 97, *P* = 0.011), particularly in single infected animals (*χ*^2^ = 57.271, *df* =8, *P* = 0.007).

Significantly higher IL-6 mRNA levels were detected after infections with *C. suis* on SD 8 (*F*_(1, 48)_ = 29.46, *P* < 0.001) and 15 (*F*_(1, 48)_ = 7.11, *P* = 0.010) when compared to the control group, as shown in Fig. 2. Among the infected groups, SL displayed the most notable increase in expression after SD 1, peaking at SD 15. SH, TL and TH showed a similar but less pronounced expression pattern. IL-6 mRNA levels of group C remained stable over time.

IL-27 mRNA levels did not differ between infected groups but were significantly higher in infected animals than in group C on SD 29 (*F*_(1, 48)_ = 12.81, *P* < 0.001). This, however, largely resulted from a significant decrease of expression in uninfected animals (Fig. 2).

Infected animals had significantly elevated IL-4 mRNA levels on SD 8 as compared to group C (*W* = 333, *P* < 0.001) while on SD 15 this was only seen in animals that received a single infection (*χ*^2^ = 38.702, *df* = 8, *P* = 0.039). They showed a significant increase of expression over time, with a peak on SD 15; trickle infected animals showed a similar although not significant pattern (Fig. 2).

On SD 8, IL-10 mRNA expression levels of infected animals were significantly higher than those of group C (*W* = 324, *P* < 0.001) (Fig. 2). Afterwards, particularly trickle infected animals experienced a further increase of IL-10 mRNA expression towards SD 29. Consequently, they expressed significantly higher levels at the end of the study compared to single infected (*χ*^2^ = 58.215, *df* = 8, *P* = 0.004) and uninfected animals (*χ*^2^ = 58.215, *df* = 8, *P* = 0.002).

TGF-β expression experienced a notable decrease after single infections; it reached lowest levels by SD 8, and then slowly increased to reach initial values by SD 29. Trickle infected animals showed a similar pattern but expression returned to initial values earlier. Single infected animals expressed significantly less TGF-β mRNA on SD 8 (*χ*^2^ = 53.346, *df* = 8, *P* = 0.015) and on SD 15 compared to control animals (*χ*^2^ = 53.346, *df* = 8, *P* = 0.002) and trickle infected animals (*χ*^2^ = 53.346, *df* = 8, *P =* 0.026) (Fig. 2). IL-1β mRNA expression was not significantly altered by infection with *C. suis* (Fig. 2).

#### Lymphatic organs

In MLN, cytokine mRNA levels were mostly unaltered upon infection, with the exception of IL-10 and TNF-α; both cytokines showed significantly elevated expression levels in infected animals (*W* = 255, *P* = 0.046 and *W* = 257, *P* = 0.040, respectively). In spleen samples, however, no group differences were found (data not shown). IL-2 mRNA levels were too low to be measured in a large number of spleen samples; it was thus not considered for statistical analyses.

#### Responders

In order to avoid animals with generally low immune responses (so called low-responders) masking differences in cytokine expression between groups and over time, statistical analyses were repeated after removing those individuals from the datasets. The allocation to responders or low-responders was based on the animals’ antibody titers, and low-responders were defined as animals with no or only a mild (less than two titration steps) increase of IgG titers against merozoites within three weeks post-infection. Due to the reduced sizes of groups SL, SH, TL and TH in this dataset, animals were categorized in uninfected and infected or single and trickle infected groups for statistical comparisons. However, results did not differ tangibly from those analyses involving all animals; they were either identical or highly similar (IFN-ɣ, IL-2, IL-6, IL-27, IL-1β, IL-4, IL-10, TNF-α), or originally significant differences disappeared after removing low-responders (IL-12, TNF-α, TGF-β) (data not shown).

### Correlations between cytokine and antibody response and parasitological parameters

The titer development of IgA against merozoites from SD 1 to 22 and SD 1 to 29 showed significant positive correlations with the IL-4 mRNA expression of SD 8 and SD 15. Positive correlations were also detected between the increase of IgG titers over time and the IL-10 expression on SD 8. The increase of IgA titers against sporozoites was positively correlated with the IL-10 mRNA expression on SD 15. When comparing expressions and titers of same study days, a significant positive correlation was found for IgA against sporozoites and IL-10 on SD 15; IgG against merozoites was positively but not significantly correlated with IL-4 mRNA levels on SD 8 (for details see Table 5). The number of days with oocyst excretion featured significant positive correlations with the IL-1β mRNA expression of SD 15 and 29 (*ρ* = 0.388, *P* = 0.006 and *ρ* = 0.446, *P* = 0.002, respectively) and the IL-4 mRNA level of SD 15 (*ρ* = 0.336, *P* = 0.018). On SD 8, a positive but not significant trend was observed for IL-1β, TNF-α and IL-10.

**Table 5:** Relationships between serum antibody titers and mRNA expression of IL-4 and IL-10. Relationships are given as Spearman’s rank correlation coefficient *ρ* (in parentheses: *P*-values). Expression on single study days was correlated with IFAT results of the same study days and with titer development over time (SD 1 to 22 and 29, respectively). Only study days with at least one significant effect are shown. Significant correlations (*P* ≤ 0.05) are indicated in bold

		IL-4		IL-10	
	SD	8	15	8	15
IgG against merozoites	8	0.259 (0.072)	**/**	0.133 (0.361)	**/**
	15	**/**	0.222 (0.121)	**/**	-0.000 (0.999)
	1–22	0.192 (0.187)	0.149 (0.301)	0.554 (**< 0.001**)	0.269 (0.059)
	1–29	-0.000 (0.999)	0.008 (0.957)	0.393 (**0.005**)	-0.002 (0.989)
IgA against merozoites	8	0.064 (0.661)	**/**	0.105 (0.472)	**/**
	15	**/**	0.049 (0.736)	**/**	0.056 (0.701)
	1–22	0.317 (**0.027**)	0.264 (0.064)	0.231 (0.110)	0.026 (0.858)
	1–29	0.374 (**0.008**)	0.370 (**0.008**)	0.151 (0.301)	-0.065 (0.654)
IgG against sporozoites	8	0.200 (0.168)	**/**	-0.106 (0.467)	**/**
	15	**/**	0.066 (0.649)	**/**	-0.182 (0.206)
	1–22	0.132 (0.364)	0.047 (0.748)	0.298 (**0.038**)	0.162 (0.262)
	1–29	0.218 (0.133)	0.131 (0.365)	0.218 (0.132)	0.250 (0.079)
IgA against sporozoites	8	0.139 (0.341)	**/**	0.188 (0.197)	**/**
	15	**/**	0.214 (0.135)	**/**	0.286 (**0.044**)
	1–22	0.220 (0.129)	0.104 (0.471)	0.258 (0.074)	0.349 (**0.013**)
	1–29	0.120 (0.410)	0.169 (0.240)	0.243 (0.093)	0.253 (0.076)

*Abbreviation*: *SD* study day

### Stimulation of lymphocytes

**Fig. 3:**
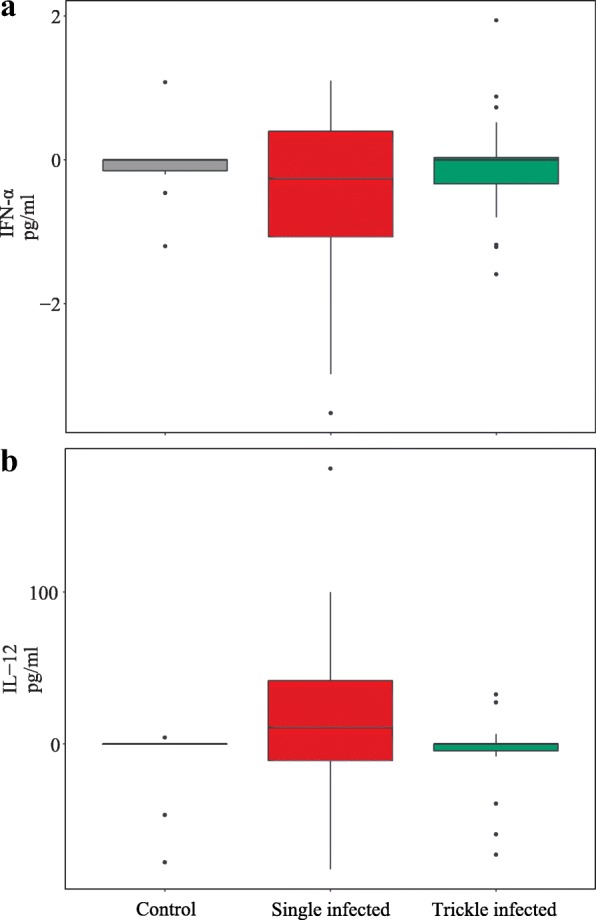
Mean IFN-α (**a**) and IL-12 (**b**) concentrations in supernatants of stimulated lymphocytes. MLN-derived lymphocytes (extracted on SD 29) were stimulated with rCSUI_005805 [44]. Adjusted values (cytokine concentrations of unstimulated controls were subtracted from those of stimulated cells) are given. Length of boxes: interquartile range; embedded line: median. Whiskers extend to the largest and smallest values within 1.5 times the length of the interquartile range from the upper and lower quartiles, respectively. Data lying outside this range are plotted as dots

Lymphocytes isolated from MLN were stimulated with a recombinant merozoite protein [44] and concentrations of various Th1, Th2, inflammatory and regulatory cytokines and chemokines in supernatants were measured. Levels of IL-10, IL-4 and IL-1β were either not detectable or very low in both infected and uninfected animals. While a production of the type II interferon IFN-ɣ could not be measured after stimulation of cells from most infected and control animals, levels of type I IFN-α were decreased in 52.5% of infected animals after antigen-stimulation. Cells of the remaining infected animals either did not respond (17.50%) or showed a negligible increase (30%). Overall, mean concentrations of IFN-α were thus reduced in supernatants of infected animals compared to those of control animals, particularly after single infections (Fig. 3a); however, differences were not statistically significant. Levels of IL-12 showed high variation between individuals, with reduced and elevated concentrations within the same group. Nonetheless, overall mean IL-12 levels were higher after stimulation of cells from infected animals; however, differences were not significant although a trend could be observed (*χ*^2^ = 4.751, *df* = 2, *P* = 0.093). This effect is largely attributed to animals which received a single infection where 65% responded with an increase of IL-12 whereas levels of trickle infected animals were mostly decreased or remained unchanged (40% each) (Fig. 3b). Levels of IL-8 were not detectable in the majority of samples; nevertheless 22.50 % of infected animals responded to antigen-stimulation with increased production of IL-8 (mean: 16.55 pg/ml). Stimulation of cells from infected animals induced an increase of CCL-2 production in 32.50% of the samples (mean: 152.30 pg/ml) and a mild decrease in only 10 %.

### Presence of *C. suis* DNA in organs

In order to examine intestinal and extraintestinal tissue for stages of *C. suis*, small intestinal samples, MLN and spleen tissue of all 50 animals were analyzed for the presence of *C. suis* DNA utilizing qPCR; however, no parasite material could be detected in any of the samples.

## Discussion

The aim of the present study was to further characterize the antibody and cytokine response of immune-competent animals undergoing an infection with *C. suis*. Understanding and eventually being able to influence the immune response of immunologically mature animals is an important step towards developing a passive immunization strategy for piglets. Previous studies indicate that the infection of immune-competent pigs induces an immune response [29, 41] but data on this age group are limited, particularly on cytokine responses. Furthermore, different protocols of oral antigen delivery were evaluated for the intensity and character of immune response they induce in order to identify a potential immunization model for sows which can improve passive protection of their offspring. Growers were chosen for this study because the pig’s immune system is fully developed at that age and immune responses should therefore be comparable to those of sows, whilst being easier to keep than sows. Since antibody levels in blood serum and colostrum of pregnant sows correlate positively [28, 29], we assume that serum titers are representative for those in colostrum and that results obtained from growers can thus be conferred to sows.

Coproscopical and serological findings confirmed that infection and a subsequent systemic immune response were successfully induced by all infection models whereas infection did not lead to clinical symptoms. Oocyst excretion was observed in every group except the uninfected control group although numbers of shedders, excreted oocysts and excretion days were low compared to experimental infections in suckling piglets [13]. The absence of clinical signs and low excretion levels are a regular phenomenon in older pigs infected with *C. suis* [1]. This lack of susceptibility may be attributed to natural age resistance as a result of the matured immune system or to the development of immunity after an overcome infection [1, 7, 47]. In fact, specific serum antibodies were, although at low levels, present in all animals at the beginning of the study, suggesting previous contact with *C. suis*. The experimental infections performed here should therefore be considered as boosters; nevertheless, they allow for an investigation of the immune response of immune-competent animals to *C. suis*, particularly when modeling the immunization of sows because an exposure of sows to *C. suis* previous to immunization is also most likely. Another explanation for the presence of titers could be the existence of extraintestinal stages [48]. To test this theory, we screened MLN and spleen samples for parasite DNA, but no *C. suis* specific DNA was detected. This does not, however, disprove the existence of extraintestinal stages. Screening of jejunum samples for *C. suis* specific DNA remained unsuccessful as well but, looking at the low oocyst excretion level, this is not surprising. It seems that, due to age resistance or immunity, the parasite can only rarely reproduce and complete its life-cycle and infection is rapidly cleared from the intestinal tissue. Moreover, the sampling time-point (four weeks after the first infection) was presumably too late: piglets infected with *C. suis* typically excrete for about two weeks with decreasing intensity [6, 47]. In histological sections, neither parasites nor typical *C. suis*-related tissue lesions were detected, underpinning the assumption that either the parasite cannot reproduce or complete its life-cycle in the intestines of immune-competent pigs or the infection is cleared completely afterwards, and lesions are not visible anymore.

Differences between infected and uninfected animals were found for several hematological parameters and white blood cell counts; however, these alterations could not be attributed to infection status of the animals or time course, and deviations from reference ranges were minor, indicating that the infection of immune-competent animals with *C. suis* does not cause appreciable systemic hematological changes. This is in contrast to the study of Worliczek et al. [26] where leucocyte and lymphocyte counts were significantly decreased in infected suckling piglets. However, these results were obtained during the acute phase of infection and from clinically affected suckling piglets.

Both IgA and IgG against sporozoites and merozoites of *C. suis* significantly increased after infection, which is consistent with what Schwarz et al. [29] described in sows, indicating that immune responses of growers and sows are comparable and that growers can be used to mimic infections in sows. Although all of the titers measured increased after infection, those against merozoites showed the most pronounced increase. Sporozoites are the initial endogenous stages of *C. suis* and invade epithelial cells after they are released from ingested oocysts. They do not replicate but develop to advanced stages during merogony and subsequent asexual multiplication results in the generation of merozoites which destroy their host cells and invade further epithelial cells [29, 48]. Merozoites might be more immunogenic because they are more numerous and thus have a higher destructive potential and longer contact time to the host compared to sporozoites, and might thus provoke a stronger immune response. Likewise, the asexual developing stages of avian coccidia are considered to be particularly immunogenic [20, 49].

In bovine eimeriosis, the development of immunity is known to be directly correlated to the primary infection dose [22]. In this study, however, only slight differences in the immune response were observed between the infection models, suggesting that the mode of infection does not noticeably affect the immunological outcome. Similarly, Worliczek et al. [47] described that the infection dose does not have a significant impact on the clinical signs or the outcome of disease in piglets, but immune responses were not looked at. Herein, titers against merozoites were significantly higher in trickle infected animals on SD 8; however, by that time all animals had just received one infection dose and differences thus have to be attributed to individual variations in the immune response. A higher IgG titer on SD 15 on the other hand might be the result of the booster infection trickle groups received on SD 8. Nonetheless, titers did not significantly differ at later time points. IgA titers against merozoites were significantly higher in group SH compared to the other infected groups at the end of the study, and a similar trend, although not significant, was observed in other titers measured. Moreover, group SH exhibited the strongest increase of all evaluated titers from SD 1 to 29 and comprised most shedders with most excretion days. This is in line with our findings that antibody titers correlate positively with oocyst excretion. Likewise, studies performed on *Eimeria* in calves, lambs and goat kids showed that IgA and IgG levels were positively correlated with oocyst excretion in most cases [30, 31, 33]. In suckling piglets infected with *C. suis*, titers and oocysts per gram of feces (OpG) were largely unrelated while titers were mostly negatively correlated with fecal consistency [28, 29]. The higher antibody production might be the result of a higher parasite reproduction rate and the completion of the life-cycle and consequently a longer contact time between antigen and immune system in the gut. The presence of intestinal IgG against merozoites supports this hypothesis since they were also positively (although not significantly) correlated with oocyst excretion and were highest in group SH. The detection of IgG in jejunal mucus of infected animals also indicates a local humoral immune response to *C. suis*; however, levels of IgA were either very low or undetectable which is in contrast to what Schwarz et al. [28] found in 28 day-old infected suckling piglets. A possible explanation could be that IgA, the primary antibody in sow milk and most relevant immunoglobulin of the local, mucosal defense [50], is primarily consumed with the milk and acts locally in the gut after the intestinal barrier becomes impermeable for antibodies rather than endogenously produced IgA, acting at the site of infection.

It has to be concluded that although antibodies seem to reflect the exposure to *C. suis* and possibly the severity of infection, they might not be correlated with anticoccidial immunity and might not contribute to protection. This is in line with the opinion of many authors [20, 22, 30, 31, 33, 51, 52] although some do suggest a protective role of (passively obtained or endogenously produced) antibodies against infection with several coccidia [23, 32, 35].

The mechanisms to successfully control coccidial infections and to develop a protective immunity are considered to be predominantly cell-mediated, and particularly characterized by the production of T helper 1 (Th1) and pro-inflammatory cytokines which are crucial in eliminating intracellular pathogens [20–24, 51]. For *C. suis* however, only few data are available on the cytokine response of the infected host, particularly on a systemic level. For this, we herein investigated the systemic cytokine profiles of unstimulated white blood cells, MLN and spleens at mRNA level and of stimulated lymphocytes at protein level. Cytokine expression levels were in part rather low, indicating that the systemic cellular immune system might not play a major role in the immune response to *C. suis*. Also, cytokine levels were subject to a high inter-individual variation which is consistent with findings of other studies on cytokine profiles in pigs [45, 53, 54] and, similar to the antibody response, differences between infected groups were mostly insignificant. Nevertheless, certain differences between infected and control animals as well as significant changes over time could be observed, particularly in white blood cells.

Expression levels of the Th1 cytokine IFN-ɣ and the inflammatory cytokine IL-2, an inducer of IFN-ɣ synthesis, decreased in white blood cells post (first) infection. TcR-ɣδ^+^ T cells are, amongst others, important producers of these cytokines [55–57] and were not only shown to be a highly abundant T cell subset in pigs but also to be significantly increased in the jejunum of *C. suis* infected piglets while numbers were reduced in blood and MLN [26, 27]. This suggests a migration of this subset of T cells to the site of infection and a predominantly local relevance of IFN-ɣ and IL-2. Local production of these cytokines, particularly of IFN-ɣ, and their essential role in the immune response against avian and various mammalian coccidia has been extensively described [21, 52, 58–63]. Alternatively, their production might have been inhibited by IL-4 and IL-10 which were both increasingly expressed after infection. Both IL-4, belonging to the Th2 family, and the regulatory cytokine IL-10 suppress the generation of Th1-associated and inflammatory cytokines and, on the contrary, promote Th2 response, B cell proliferation and antibody production [24, 45, 54, 64, 65]. This matches the increase of serum antibody levels observed post-infection and the significant positive relations between IL-4, IL-10 and antibody titers shown in this study, indicating a Th2-biased immune response. Increased peripheral levels of IL-4 and IL-10 have also been described in *Neospora caninum* infected cattle, but are thought to be associated with a decreased ability of the host to control infections with coccidia [52, 61, 66]. IL-10 mRNA expression was also significantly elevated in MLN of infected animals which matches findings of Worliczek et al. [26] where the number of B cells was significantly increased in MLN of infected piglets. The simultaneously increased expression of TNF-α mRNA is a commonly observed phenomenon because an elevated production of TNF-α is usually counterbalanced by IL-10 production to avoid excessive inflammatory responses [67]. However, levels of both cytokines were not drastically increased, thus these results have to be interpreted with caution. In contrast to IFN-ɣ and IL-2, mRNA expression of IL-12p35, a subunit of the Th1-associated cytokine IL-12, was significantly increased on SD 8 after infection. This is surprising since IL-12 is an important enhancer of IFN-ɣ production [64], thus one would expect a simultaneous increase of both cytokines, and furthermore IL-10 usually downregulates IL-12 [65]. However, inter- and intra-individual variation of IL-12p35 mRNA expression was high and also observed in group C, and differences were only slightly significant (*W* = 281, *P* = 0.032). Moreover, IL-12 is known to be regularly produced by a broad range of cells in humans and animals [64, 68], and the changes observed might be attributed to physiological fluctuation or induced by other stimulators than *C. suis*. Similarly, mRNA expression of inflammatory TNF-α was subject to some variation in white blood cells of both infected and uninfected animals; group differences rather resulted from varying expression levels in group C, although levels also slightly increased over time in infected animals. TNF-α is, in part, produced by TcR-ɣδ^+^ T cells and its expression was shown to be increased in the jejunum of infected piglets [27], thus it might be more relevant and consequently TNF-α producing cells might be more abundant at the site of infection. Expression of TGF-β was significantly downregulated in infected animals in the first two weeks post-infection. Similarly, local TGF-β expression was found to be downregulated in *N. caninum* infected cattle and sheep [52, 63] and unaltered in the jejunum of *C. suis* infected piglets [27]. TGF-β is a regulatory cytokine which suppresses Th1 and inflammatory cytokine production in order to avoid excessive inflammatory responses with tissue-damaging consequences [60, 63]. Since IL-10 mRNA expression was increased in this study, one might speculate that *C. suis* primarily activates the production of IL-10 by regulatory T cells rather than that of TGF-β. Alternatively, TGF-β expression could be suppressed in favor of a local Th1-biased immune response which would explain the severe damage of the intestinal mucosa in the course of *C. suis* infections in suckling piglets since TGF-β is involved in its protection [60]. However, more data on cytokine expression in the jejunum are needed to test this theory. The increased mRNA expression of IL-6 on SD 8 and 15 indicates an acute-phase response to infection with *C. suis*. Increased serum and local levels of IL-6 were reported in humans, mammals and birds after infections with coccidia [52, 62, 63, 69, 70]. In addition, IL-6, secreted in vitro by bovine fibroblasts infected with *N. caninum*, induced the production of IL-17 by T cells which in turn significantly reduced parasite burden [71]. Since IL-6 can also promote a humoral immune response [52], together with IL-4 and IL-10 it may be responsible for the antibody increase observed in this study.

In vitro stimulation of MLN-derived lymphocytes of infected animals with a recombinant merozoite protein resulted in increased levels of IL-12, supporting other studies on coccidia [51, 66, 70] and the assumed key role of a Th1-biased immune response in the successful elimination of protozoa. However, protein levels were low with very high variations, and differences between infected and control animals did not reach significant levels. Also, other cytokines frequently reported to be produced upon stimulation with coccidial antigens were not increased. Potentially, the in vitro application of the protein alone does not sufficiently stimulate the immune cells due to a weak immunogenicity which is also a common issue in vaccines based on recombinant proteins unless combined with immunostimulants [72]. Of the interferon family two members were measured: the type II interferon IFN-ɣ is widely associated with controlling coccidial infections [24, 73], but an antigen-induced production could not be observed in this study. Production of IFN-α, a representative of type I interferons, was (although not significantly) reduced in cells of infected animals. IFN-α is primarily recognized for its antiviral activity [73] and has only been studied to a limited extent in the context of coccidial infections; however, it was shown to limit parasite growth in cells infected with *N. caninum* and other coccidia [73, 74]. *Toxoplasma gondii* was shown to be able to inhibit IFN-α production by host cells [73] and *C. suis* might possess the same ability in order to escape parasite-limiting immune responses. As mentioned above, the changes observed are mostly rather small, since protein levels appeared very low or were not measurable at all in many cases, and warrant cautious interpretation. Further attempts should be made to improve this assay.

## Conclusions

Overall, our findings indicate that the infection of immune-competent animals with *C. suis* does induce a systemic immune response, irrespective of infection dose or frequency, without clinical signs and with negligible oocyst excretion. Immune responses of sows and growers can therefore be considered as comparable, thus they can be used as models to mimic infection or immunization of sows with this parasite. Moreover, the induction of an immune response without clinical signs or oocyst excretion is an ideal situation for a passive vaccination strategy. However, the different modes of oral infection tested here did not significantly differ in their immunological outcome, thus application routes other than the oral one, the use of adjuvants, or the use of recombinant or DNA vaccines should be taken into consideration to enhance immune responses and subsequently improve passive protection for piglets. Systemic immune responses were associated with upregulated Th2 and regulatory cytokines and increased serum levels of *C. suis*-specific antibodies whereas Th1-associated cytokines typically involved in coccidian infections were downregulated or unaffected by infection. Generally, systemic cytokine responses were weak and showed considerable variation within groups. However, unlike certain *Eimeria* species, *C. suis* reproduces solely within the superficial layers of the intestinal mucosa, thus the strongest cellular immune reactions, particularly those of the Th1 and proinflammatory type, might take place at the site of infection where the parasite has direct contact to local immune cells. It is conceivable that certain cytokine profiles observed in the blood are simply a spillover effect of the local immune response. Finally, none of the measured parameters could be clearly identified as a marker related to protection. Antibody titers were positively correlated with oocyst excretion, with IgG titers against merozoites exhibiting the strongest increase, resulting in highest levels; it might thus serve as a correlate for parasite replication or the severity of infection. It cannot, however, be ruled out that an enhanced immune response in sows would confer sufficient passive protection to piglets, either through colostral antibodies or cellular components or both. Since pre-experimental antibody titers indicated previous infections of the growers with *C. suis*, the reactions to the experimental infections must be seen as immune memory response (which would mimic the situation in sows), although from the generally low responses in most pigs this could not be determined unequivocally.

## Additional file

Additional file 1:**Table S1.** Hematological parameters and white blood cell counts determined on study days 1 and 29. *Abbreviations*: MCV, mean cell volume; MCH, mean cellular hemoglobin; MCHC, mean cellular hemoglobin concentration. **Table S2.** Differences in hematological parameters and white blood cell counts between groups and over time. Values of SD 1 and SD 29 were compared between uninfected animals and infected animals altogether. Additionally, differences over time were calculated for both groups. *Abbreviations*: $\overline{\mathrm{x}}$, mean; σ, standard deviation. **Table S3.** Serum titer development over time within groups. In brackets: test statistic *χ*^2^ and degrees of freedom *df* according to the Friedman rank sum test. *Abbreviations*: SD, study day; $\overline{\mathrm{x}}$, mean titer; σ, standard deviation. **Table S4.** Results (as *P*-values) of antibody titer comparisons between groups on each study day. Significant differences (*P* ≤ 0.05) are indicated in bold. *Abbreviations*: SD, study day; SL, Single Low; SH, Single High; TL, Trickle Low; TH, Trickle High; C, Control. **Table S5.** Change of cytokine mRNA expression of white blood cells over time within groups. Expression levels of study days 8, 15 and 29 of each group were compared to those of study day 1. Significant differences (*P* ≤ 0.05) are indicated in bold. *Abbreviations*: SD, study day; SL, Single Low; SH, Single High; TL, Trickle Low TH, Trickle High; C, Control. **Table S6.** Cytokine mRNA expression of white blood cells before infection. Expression values of study day 1 were compared between groups and resulting *P*-values are shown. Significant differences (*P* ≤ 0.05) are indicated in bold. *Abbreviations*: SL, Single Low; SH, Single High; TL, Trickle Low; TH, Trickle High; C, Control. (XLSX 34 kb)

## Abbreviations

BW: Body weight
FCS: Fetal calve serum
FMIA: Multiplex fluorescent microsphere immunoassay
IFAT: Immunofluorescence antibody test
IFN: Interferon
Ig: Immunoglobulin
IL: Interleukin
IPC: Interplate calibrator
IPEC: Intestinal porcine epithelial cells
MLN: Mesenteric lymph nodes
mRNA: Messenger ribonucleic acid
OAZ: Ornithine decarboxylase antizyme
PBS: Phosphate buffered saline
PCR: Polymerase chain reaction
RPL4: Ribosomal protein L4
SD: Study day
TGF: Transforming growth factor
